# Research note: Application of convolutional neural networks for feather classification in chickens

**DOI:** 10.1016/j.psj.2025.105254

**Published:** 2025-05-02

**Authors:** Jiajia Niu, Tong Li, Kunlong Qi, Yang Liu, Haixuan Deng, Yujia Hu, Dan Xu, Liuting Wu, Felix Kwame Amevor, Yingjie Wang, Gang Shu, Xiaoling Zhao

**Affiliations:** a State Key Laboratory of Swine and Poultry Breeding Industry, College of Animal Science and Technology, Sichuan Agricultural University, Chengdu, Sichuan, PR China; b Key Laboratory of Livestock and Poultry Multi-omics, College of Animal Science and Technology, Sichuan Agricultural University, Chengdu, Sichuan, PR China; c Farm Animal Genetic Resources Exploration and Innovation Key Laboratory of Sichuan Province, College of Animal Science and Technology, Sichuan Agricultural University, Chengdu, Sichuan, PR China; d Department of Basic Veterinary Medicine, Sichuan Agricultural University, Chengdu, Sichuan 611130, PR China

**Keywords:** Feather color, Convolutional Neural Network, chicken, breeding, classification

## Abstract

Feather color plays a crucial role in distinguishing various poultry breeds. However, the reliability of conventional manual classification methods is debated due to the intricate nature of feather color traits. To address this issue, we applied Convolutional Neural Networks (CNN) to extract the feather texture features of 300 images each of Golden Meihua (GM) and Silver Meihua (SM) feathers. The nonlinear features were then learned through an MLP layer and activation functions to complete the classification of GM and SM. Finally, we successfully developed a method automating the identification of feather texture features, achieving a recognition model accuracy of 93.71 % after 5-fold cross-validation. This research improves the precision and effectiveness of feather color selection, offering valuable insights into the systematic classification of feather colors in diverse poultry species.

## Introduction

Feather color is a vital trait in poultry breeding, serving as a key factor in distinguishing breeds and aiding breeders in selecting superior genetic sources for enhancement (Andersson et al., 2020; Jingyi, et al., 2021). Beyond its aesthetic value, feather color significantly influences genetic research, economic performance, and production efficiency, highlighting the need for accurate and timely identification. Poultry exhibit extensive feather color variations, including differences in pigment depth and type (dark eumelanin and red/yellow pheomelanin) as well as structural coloration (Andersson, et al., 2020). This diversity is further enriched by unique patterns found on various body parts or even within individual feathers. In domestic chickens, eight primary feather patterns have been identified: stippling, autosomal barring, sex-linked barring, penciling, single lacing, double lacing, spangling, and mottling (Andersson, et al., 2020; Jingyi, et al., 2021). Feather color composition is complex, with non-uniform coloration often observed within the same feather or across feathers from different body parts. This complexity poses significant challenges in establishing a universally applicable definition for poultry feather color classification. Conventional techniques such as visual scoring (Cruz-Bernate, et al., 2023) and spectrophotometry (Romero-Diaz, et al., 2022) are commonly utilized in feather classification due to their accessibility and cost-effectiveness. However, these methods present substantial limitations when applied to the analysis of intricate feather patterns. Visual scoring is inherently subjective, prone to observer bias, and exhibits limited reproducibility, while spectrophotometry, although precise in quantifying color, is restricted to uniform surface measurements and lacks the capacity to discern spatial or textural heterogeneity (Romero-Diaz, et al., 2022). These methodological shortcomings underscore the need for more sophisticated, automated analytical frameworks particularly those capable of capturing complex, non-uniform color distributions and fine-scale feather texture with minimal human input.

**Fig. 1:**
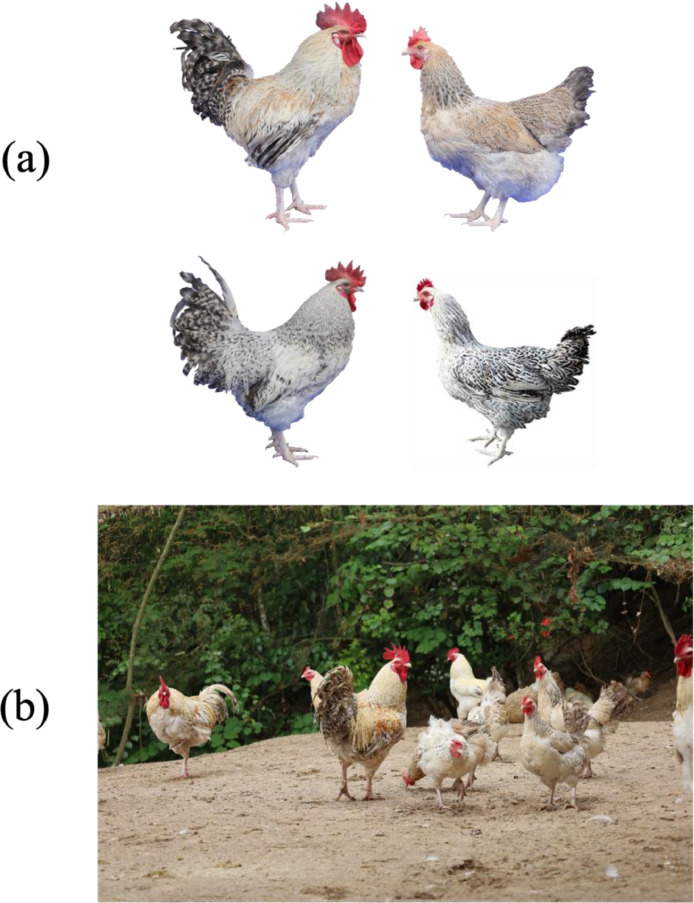
Shan Di Mei Hua chicken feather phenotype. (a) Golden Meihua (GM) and Silver Meihua (SM) Individuals (up is GM, down is SM). (b) The Shan Di Mei Hua chicken group.

The Shan Di Mei Hua chicken, a unique breed native to China, is renowned for its distinctive feather colors. This breed primarily exhibits two plumage types: Golden Meihua (GM) and Silver Meihua (SM) (Fig. 1**a**). However, intermediate feather colors are also observed (Fig. 1**b**), complicating visual classification. Traditional methods rely heavily on subjective human judgment, which can undermine the precision and reliability of classification. Recent advances in deep learning, particularly convolutional neural networks (CNNs), have revolutionized image classification and object detection, offering superior performance over conventional pattern recognition techniques (Nithya, et al., 2023). CNNs effectively extract intricate features from images, making them valuable for applications such as health monitoring (Chen, 2021) and behavior recognition (Fang, et al., 2021). Despite these advancements, CNNs have not yet been applied to poultry feather recognition. Like EEG-based diagnostics that utilize structural pattern differences to distinguish between depressive and healthy states (Özbek et al., 2024), our study applies CNN to capture and classify subtle textural features in feather images for accurate breed identification. This study aims to leverage CNNs to extract texture features from GM and SM feathers and develop an automated classification system capable of accurately recognizing feather colors. This approach seeks to overcome the limitations of traditional, subjective methods and provide a reliable tool for feather color-based breeding selection, ultimately enhancing poultry breeding practices.

## Materials and methods

### Image collection and pre-processing

**Fig. 2:**
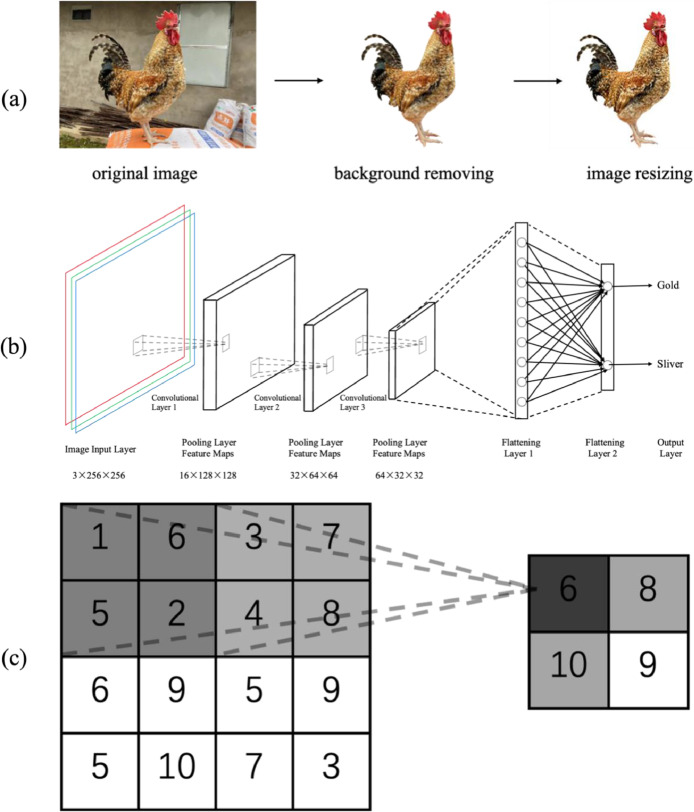
Model construction process. (a) Image Pre-processing. (b) Convolutional Neural Network Architecture. Convolutional layer 1: input = 3 × 256 × 256, output channels = 16, kernel size = 3, padding = 1; Pooling layer 1: kernel size = 2, stride = 2; Convolutional layer 2: input = 16 × 128 × 128, output channels = 32, kernel size = 3, padding = 1; Pooling layer 2: kernel size = 2, stride = 2; Convolutional layer 3: input = 32 × 64 × 64, output channels = 64, kernel size = 3, padding = 1; Pooling layer 3: kernel size = 2, stride = 2 (c) Max pooling.

*In this study, the images of the adult Shan Di Mei Hua chickens were captured using a Canon EOS RP mirrorless camera with a resolution of 6240 × 4160 pixels at a cage-rearing farm in Ya’an, Sichuan, China. A total of 400 chickens were randomly selected, evenly split into Golden Meihua (GM) and Silver Meihua (SM) groups, with equal representation of males and females. At least 300 images per group were collected from various angles and postures, stored in JPG format (3024 × 4032 pixels). To reduce noise caused by environmental factors, median filtering was applied. Backgrounds were removed using the rembg library, and im-ages were resized to 256 × 256 pixels for consistency in feature extraction and analysis* (Fig. 2a).

### Dataset construction

The pre-processed image data was organized in a PyTorch-compatible format and classified using the `datasets.ImageFolder` method for efficient loading. Data augmentation and normalization were applied through the `transforms.Compose` function, which enhanced the effectiveness of model training.

### Model architecture

A convolutional neural network (CNN) was used as the base model to extract features and classify the 480 images of Shan Di Mei Hua chickens. The network architecture consists of three convolutional layers, three pooling layers, and two fully connected layers (Fig. 2**b**). Dropout layers were incorporated to prevent overfitting and improve the model's ability to generalize. The first layer is Convolutional and Pooling Layers. The primary function of the convolutional layers is to extract features from the images. Each convolutional layer consists of a set of learnable filters (also known as kernels). During the network’s forward pass, each kernel performs convolution operations with the input data at a specified stride, producing a feature map for that layer after applying a nonlinear activation function. The feature map generated by a convolutional layer can be represented as follows:

$$\text{Hout}=\lfloor \frac{\text{Hin}+2\;\times \;\text{Padding}-\text{KernelSize}}{\text{Stride}}\rfloor +1$$

$$\text{Wout}=\lfloor \frac{\text{Win}+2\;\times \;\text{Padding}-\text{KernelSize}}{\text{Stride}}\rfloor +1$$

where Hin and Win are the input feature map’s height and width, Hout and Wout are the output feature map’s height and width, Padding is the padding size, KernelSize is the filter size, and Stride is the stride size. Pooling layers reduce the dimensionality of the feature maps by aggregating values within a region and mapping them to a single value, thereby reducing the feature map size. Max pooling, the most commonly used pooling method, was employed (Fig. 2**c**). The second layer is Fully Connected Layers. The fully connected layers convert the multi-dimensional feature maps into one-dimensional vectors while preserving essential information. In a CNN architecture used for image classification, the fully connected layers are typically placed at the end of the network, where they feed the extracted features into the classifier. In this study, Fully Connected Layer 1 takes an input of 64 × 32 × 32 and outputs 512 neurons, while Fully Connected Layer 2 takes 512 neurons as input and outputs 2 neurons. The third layer is Classification Layer. During training, the classification layer calculates the error between the predicted values and the true labels of network. This error is minimized through backpropagation and gradient descent to reduce the loss of model. During testing, the classification layer computes the probability that the sample image belongs to each category and assigns it to the category with the highest probability. The most commonly used classifier in CNNs is Softmax regression. In this study, the classification layer has two final categories: GM and SM.

### Model training

To evaluate the model performance, 5-fold cross-validation was applied using 60 images of Shan Di Mei Hua chickens. K-fold cross-validation is a model evaluation technique that estimates the performance of a machine learning model by dividing the dataset into K equal-sized subsets (folds) and performing K iterations of training and validation. In each iteration, one subset serves as the validation set, while the remaining K-1 subsets are used for training. This process ensures that every sample is used as a validation set at least once, leading to a more reliable performance evaluation. The final model performance is represented by the average result of the K validations, thus reducing uncertainty due to data splitting. In this study, 5-fold cross-validation was used. In each fold, the Adam optimizer and cross-entropy loss function were employed to train and optimize the model, with the goal of improving classification accuracy. During model training, the Adam optimizer was used to update the model parameters. Adam (Adaptive Moment Estimation) is an adaptive learning rate optimization algorithm that combines the advantages of momentum and RMSProp. It maintains an adaptive learning rate for each parameter by combining first-moment (mean) and second-moment (variance) estimates of the gradients. The optimizer computes these estimates and applies bias correction to both, adjusting the learning rates accordingly. This method promotes good convergence and stability during training, making it well-suited for optimizing deep learning models. The cross-entropy loss function was employed to evaluate the classification performance of the model. For binary classification, the formula used is:

Loss=−(ylog(p)+(1−y)log(1−p))

This function measures the difference between the predicted probability distribution and the true label distribution. A smaller loss value indicates better model performance, and continual optimization of the loss function improves model performance to accurately classify GM and SM.

All the code used in the method can be downloaded at: https://github.com/laviniaguci/feather-color.git.

## Results and discussion

In this study, the performance of a Convolutional Neural Network (CNN)-based approach for chicken feather classification was evaluated in comparison to traditional classification methods. Conventional techniques, such as visual scoring (Cruz-Bernate, et al., 2023) and spectrophotometry (Romero-Diaz, et al., 2022), have been widely employed; however, they exhibit notable limitations in processing complex feather patterns and rely heavily on subjective human input. In contrast, the CNN model employed in this study effectively mitigates these constraints by autonomously extracting and learning discriminative features from image data. A total of 300 feather images per group were utilized, systematically partitioned into training (80 %), testing (10 %), and validation (10 %) sets to ensure model robustness and generalizability. The CNN model achieved a classification accuracy of 93.71 % on the test set of 60 images, demonstrating the robustness of the model. Consistency across the folds highlighted the reliability of the methodology. Despite the high accuracy, some classification errors occurred, attributed to factors such as low image resolution, complex acquisition conditions (Fang, et al., 2021), insufficient sample size, and suboptimal imaging equipment (Wang, et al., 2023). Previous studies applying machine learning to poultry phenotype classification have encountered challenges related to environmental variability, background interference, and computational constraints, all of which can affect model accuracy. For instance, Fang et al. (2021) used a deep neural network to classify broiler behaviors and reported high accuracy for easily distinguishable behaviors like eating (93.61 %) and resting (96.23 %), but significantly lower accuracy for walking (51.35 %) and running (62.70 %) due to the similarity of postural features. These findings underscore the complexity of phenotypic image recognition and the need for more robust algorithms to improve classification precision under variable conditions. These results emphasize the importance of optimizing segmentation thresholds, improving feature extraction, and exploring more precise recognition parameters. Future studies should improve recognition accuracy by expanding datasets across breeds and growth stages, utilizing multi-angle imaging, and optimizing algorithms for greater stability. Integrating non-contact monitoring and advanced imaging tools such as color, infrared, and 3D cameras will further enhance data precision and phenotypic analysis (Wang, et al., 2023). Environmental conditions, such as temperature and dust, significantly affect equipment performance, highlighting the importance of using durable, high-resolution devices and conducting regular maintenance. Limitations in equipment setup, such as fixed camera angles and lighting conditions, also impact model accuracy (Li et al., 2021). Many studies focus on specific body parts, like the head or torso, neglecting areas such as wings and tails. Advancing mobile image recognition technologies and segmentation algorithms may enable detailed data collection on individual poultry, improving overall classification accuracy. In conclusion, this study successfully applied CNNs to classify feather characteristics in Shan Di Mei Hua chickens. By leveraging advanced techniques, including 5-fold cross-validation, the Adam optimizer, and the cross-entropy loss function, the model achieved high classification accuracy. However, the study’s focus on a single breed and growth stage, as well as the limited dataset size, poses challenges to its generalizability. Expanding datasets and refining algorithms could improve performance and broaden the applicability of this approach. These findings provide valuable insights for poultry breeding programs and lay the foundation for future advancements in machine learning-based phenotype recognition.

## Ethics approval

Not applicable.

## CRediT authorship contribution statement

**Jiajia Niu:** Conceptualization, Data curation, Formal analysis, Methodology, Writing – original draft. **Tong Li:** Formal analysis, Software, Methodology. **Kunlong Qi:** Formal analysis, Software, Methodology. **Yang Liu:** Conceptualization, Data curation, Formal analysis, Supervision, Writing – review & editing. **Haixuan Deng:** Conceptualization, Data curation, Formal analysis, Supervision, Writing – review & editing. **Yujia Hu:** Conceptualization, Data curation, Formal analysis, Supervision, Writing – review & editing. **Dan Xu:** Conceptualization, Data curation, Formal analysis, Supervision, Writing – review & editing. **Liuting Wu:** Conceptualization, Data curation, Formal analysis, Supervision, Writing – review & editing. **Felix Kwame Amevor:** Conceptualization, Data curation, Formal analysis, Supervision, Writing – review & editing. **Yingjie Wang:** Conceptualization, Data curation, Formal analysis, Supervision, Writing – review & editing. **Gang Shu:** Conceptualization, Data curation, Formal analysis, Supervision, Writing – review & editing. **Xiaoling Zhao:** Conceptualization, Data curation, Formal analysis, Funding acquisition, Supervision, Writing – review & editing.

## Disclosures

The authors declare that they have no known competing financial interests or personal relationships that could have appeared to influence the work reported in this paper.
